# The healthcare burden imposed by children with gastroparesis and the shift imposed by COVID-19

**DOI:** 10.3389/fped.2025.1622205

**Published:** 2025-07-22

**Authors:** Christian Sadaka, Binghong Xu, Alain Benitez, Carolyn Orians, Haley Pearlstein, Hayat Mousa

**Affiliations:** ^1^ GI, Hepatology & Nutrition Department, The Children’s Hospital of Philadelphia, Philadelphia, PA, United States; ^2^ Perelman School of Medicine, University of Pennsylvania, Philadelphia, PA, United States

**Keywords:** gastroparesis, cost, healthcare utilization, category, region, feeding supplementation, functional gastrointestinal disorder, disorders of the gut-brain interaction

## Abstract

**Goals:**

We aim in this study to report the trend of annual economic burden of children admitted with Gastroparesis (GP) over the last 10 years and evaluate the possible effect of COVID-19.

**Background:**

Inpatient healthcare utilization by children with GP was last reported between 2004 and 2013. Since then, the effect of the COVID-19 pandemic has not been evaluated.

**Study:**

We used the Pediatric Health Information System (PHIS) database to retrieve data recorded in 42 children’s hospitals between January 2014 and September 2023 with GP being a primary or secondary diagnosis.

**Results:**

A total of 20,293 pediatric gastroparesis admissions were documented. The total cost was $1,323,541,518. The average admission cost was $65,222 and the median was $18,921. Reviewing the possible effect of COVID-19, we found that the highest annual mean and median costs were in 2020, and the highest annual total cost was in 2022. The costs are divided over 6 different categories: clinical, imaging, lab, pharmacy, supplies, and others, with the highest impacts resulting from these 3 categories: clinical, pharmacy, and others. The mean and median costs differ in the 4 regions, Northeast, South, Midwest and West, with the highest in the Midwest. Of all the admissions, 15.6% had a code for nasogastric tube (NG) present, 40.7% used the code for a gastrostomy tube (G-tube), 10.0% had a code for a jejunostomy tube (J-tube) and 24.6% required nutrition support via surgical feeding tubes.

**Conclusion:**

This PHIS database study confirms an upward trend in the annual healthcare utilization by children admitted with GP, resulting in an upward trend in the total economic burden on children's hospitals emphasized by the COVID-19 pandemic.

## Introduction

Gastroparesis (GP) is a chronic disorder of delayed gastric emptying in the absence of mechanical obstruction. Common upper GI symptoms include nausea, vomiting, early satiety, postprandial fullness, and abdominal pain (1, 2). GP affects patients of all ages; however, it is more common in adults (3). While diabetes is the most common contributor in adults, idiopathic cases represent the largest portion in pediatrics (1, 4–6). Database review studies have found total GP prevalence ranging from 0.014% to 0.27% of the general population with 2.5–18-fold increase in the total number of GP admissions and emergency department visits over the past 2 decades (1, 5, 6). However, the prevalence in the pediatric population is still unclear; the most recent inpatient nationwide study conducted on patients between 2004 and 2013 showed that the number of unique patients hospitalized with GP increased from 174 in 2004 to 723 in 2013 (7).

There are few studies assessing the economic impact caused by GP (2). One study found that GP admissions and their respective costs have increased 10-fold between both the 1990s and 2000s and between the 2000s and 2010s. The increase in GP cost was accompanied by a 3-fold increase in the GP proportion of the national bill utilization; the aggregate charges of all inpatient medical expenses (2, 8). Furthermore, a 3-year follow up study found that the cost of each individual GP patient remains elevated throughout the duration of the study (9). More recently, a study done by Lu and Mousa et al. analyzed the economic burden of GP admissions in pediatric hospitals between 2004 and 2013. This pioneer study found a 5.8-fold increase in total annual costs of GP admissions from 2004 to 2013, with a mean annual increase of $3.4 million (7).

The Coronavirus Disease 2019 (COVID-19) pandemic led to a global economic crisis with a huge impact on the healthcare systems (10). By September of 2021, COVID-19 accounted for around 86% of the total healthcare expenditure since the start of the pandemic costing billions of dollars (11). Direct medical costs of COVID-19 positive patients were 1.7 times and 1.46 times higher in pediatric and adult patients respectively compared to non-COVID-19 patients up to 6 months follow up (12). In addition, healthcare utilization in COVID-19 patients was found to be elevated for at least 1 year after the acute infection (13).

COVID-19 can lead to long term symptoms, such as with Long-COVID (14) and are associated with an increase in Functional GI disturbances [now known as disorders of the gut-brain interaction (DGBI)] such as Irritable Bowel Syndrome (IBS), Functional Dyspepsia (FD), and Gastroparesis (15–20). Moreover, during the pandemic, patients with DGBI had worsening symptoms, especially nausea and vomiting, and required increased healthcare usage compared to the 6 months prior to the pandemic's start (21).

In this study, we aim to report inpatient care related costs in US pediatric patients with GP from January 1, 2014, through September 30, 2023. We will describe cost distribution over different categories and areas and report the feeding supplies use and attributable costs. Finally, we evaluate the impact of COVID-19 on the cost of GP admissions.

## Methodology

### Study design

**Figure 1:**
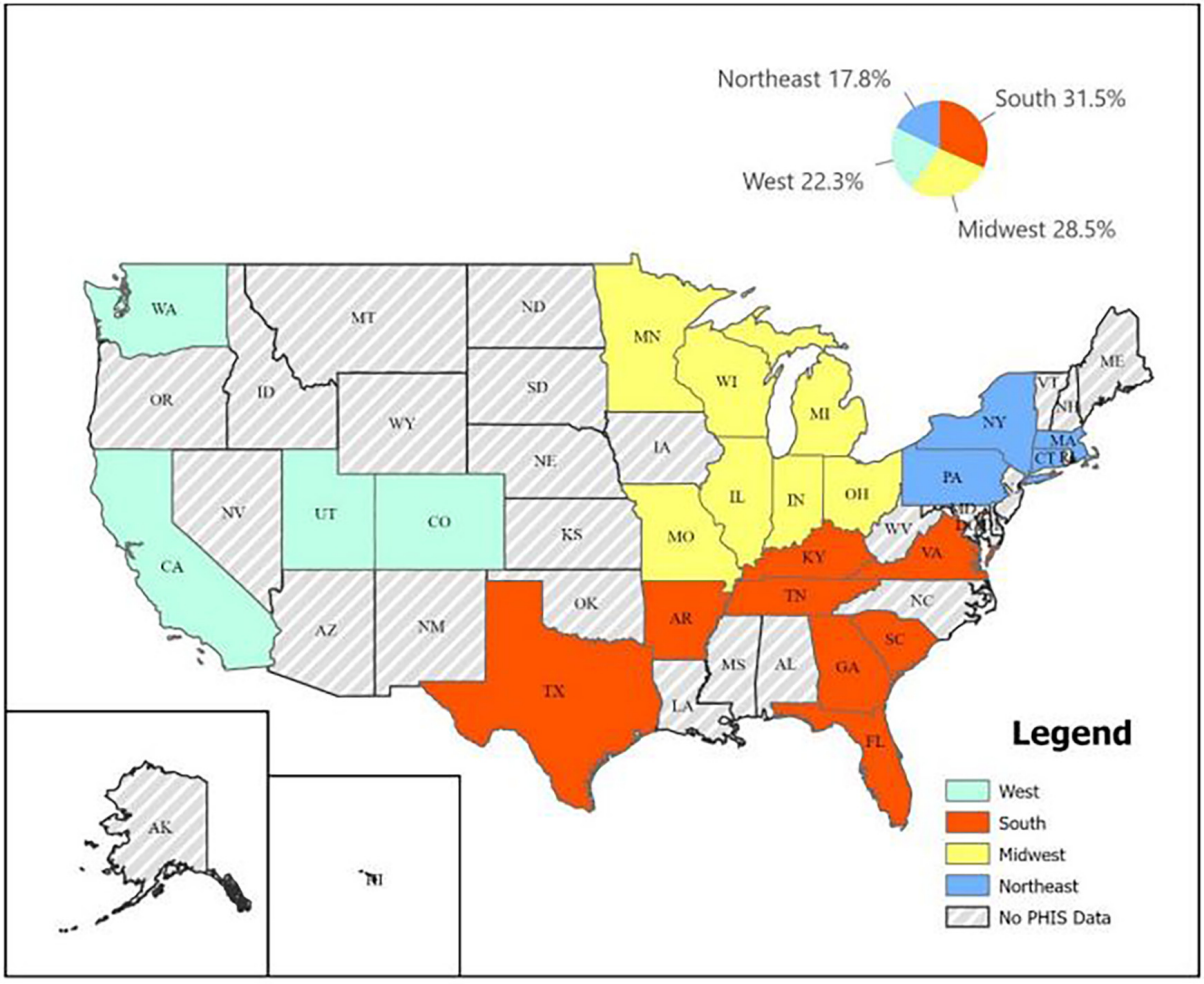
United States of America map depicting the PHIS distribution of states into different regions.

We used the Pediatric Health Information System (PHIS) database, managed by the Children's Hospital Association, to retrieve data for our retrospective case study. PHIS collects inpatient billing data from over 49 pediatric hospitals in the United States (22). We excluded 7 hospitals that did not contribute their data for the whole duration of the study, from January 1, 2014, until September 30, 2023, leaving 42 pediatric hospitals included in this study. The hospitals are divided into 4 major regions according to the state they are found in: Northeast, South, Midwest, and West. However, the number of contributing hospitals in each region varies. There are 6 children's hospitals in the Northeast region, 14 in the South, 12 in the Midwest, and 10 in the West region. Figure 1 shows the United States of America map depicting the PHIS distribution of states into different regions.

We reported data on all children with GP and included all pediatric age groups up to 25 years of age. We used the Gastroparesis ICD-9 (536.3) and ICD-10 (K31.84) codes to retrieve the data. The encounters with GP as a primary or secondary diagnosis were included. Visits with GP as a secondary diagnosis were required to have at least one of the symptoms or conditions in Table 1 as a primary diagnosis. We collected all admissions’ cost data and adjusted the numbers to accommodate for inflation according to the 2023 US dollar value. We retrieved additional patient data such as age, gender, race, and geographic location. In addition, we gathered hospitalization information including the total number of patients, admissions, the length of stay (LOS), and geographic location. We then used CPT codes found in Table 2 and PHIS specific codes to retrieve data about feeding supplementation. Feeding supplementation was not necessarily the reason for admission but was required during the admission. Data on parenteral nutrition (PN) was not easily retrievable.

**Table 1:** ICD-9 and ICD-10 required as primary diagnosis.

Diagnosis	ICD-9	ICD-10
Nausea	787.02	R11.0
Vomiting	787.03	R11.1
Nausea and Vomiting	787.01	R11.2
Generalized Abdominal Pain	789.07	R10.84
Unspecified Abdominal Pain	789.00	R10.9
Epigastric Pain	789.06	R10.13
Abdominal Distension	787.3	R14.0
Early Satiety	780.94	R68.81
Belching	787.3	R14.2
Constipation	564.0	K59.00
Rumination	307.53	F98.21
Weight Loss	783.21	R63.4
Inadequate Oral Intake	783.9	R63.8
Total Parenteral Nutrition (TPN)	V68.1	Z76.0
Obstruction of Feeding Tube	996.59	T85.598A
Avoidant-Restrictive Food Intake Disorder (ARFID)	307.59	F50.82
Malnutrition	262, 263.0, 263.1, 263.9	E43, E44.0, E44.1, E46

**Table 2:** CPT codes used to retrieve data about tube feeding.

Tube used	CPT code
Nasogastric tube	43752
Gastrostomy tube	49440, 43246
Jejunostomy tube	49446, 44373, 44186, 44015, 44300, 44372

### Outcome measures

Our primary outcome was to report the trends in healthcare utilization (cost) of pediatric gastroparesis inpatient care. We also evaluated the effect of COVID-19 pandemic on admissions costs by comparing the costs before and after the beginning of the COVID-19 pandemic. While we agree that some of the costs may be attributable to factors beyond gastroparesis (GP), we would like to emphasize that for an admission to be classified as a GP-related admission, it must include a gastrointestinal (GI)-related primary diagnosis. This criterion ensures that the GI condition—specifically GP—is the primary driver of the associated costs.

The secondary outcome was to describe patient characteristics, admission metrics in addition to the need for nutritional support used in these admissions.

### Analysis

We reported both mean and median data on costs and length of stay to highlight the possible effect of outliers contributing to prolonged hospitalization and increased costs.

The PHIS database categorizes expenses into 6 categories: Clinical, Pharmacy, Imaging, Supplies, Lab, and Others. The “Clinical” category included expenses attributed to the treating team (physician, nurse practitioner, physician assistant), therapists, and other hospital personnel. The “Pharmacy” category encompasses all expenses pertaining to medications and parenteral feeding. The “Imaging” category constitutes the cost of all forms of imaging (MRI, CT, x-ray, fluoroscopy, nuclear medicine, ultrasound) and interventional radiology. The “Supplies” category includes expenses related to the feeding, cardiovascular, orthopedic, rehabilitation, surgical, nursing and/or other medical supplies and devices. The “Lab” category constitutes the expenses of all laboratory tests done, the blood bank, microbiology, and pathology. Finally, the cost under “Others” category is mostly attributable to the floor services: nursing units, psychiatric treatment units, ancillary services to treatment, dietary services, social services, discharge planning services, and other ancillary services.

Statistical analyses were performed using SPSS version 29.0 (IBM Corporation, Armonk, NY). Variables were summarized with descriptive statistics, either as mean with standard deviation (SD) or median and interquartile range (IQR). Statistical comparisons were performed between study groups using Student's *t*-test, chi-squared tests, Mann–Whitney *U*, Kruskal–Wallis, or Fisher's exact tests as appropriate.

## Results

### Demographics

**Figure 2:**
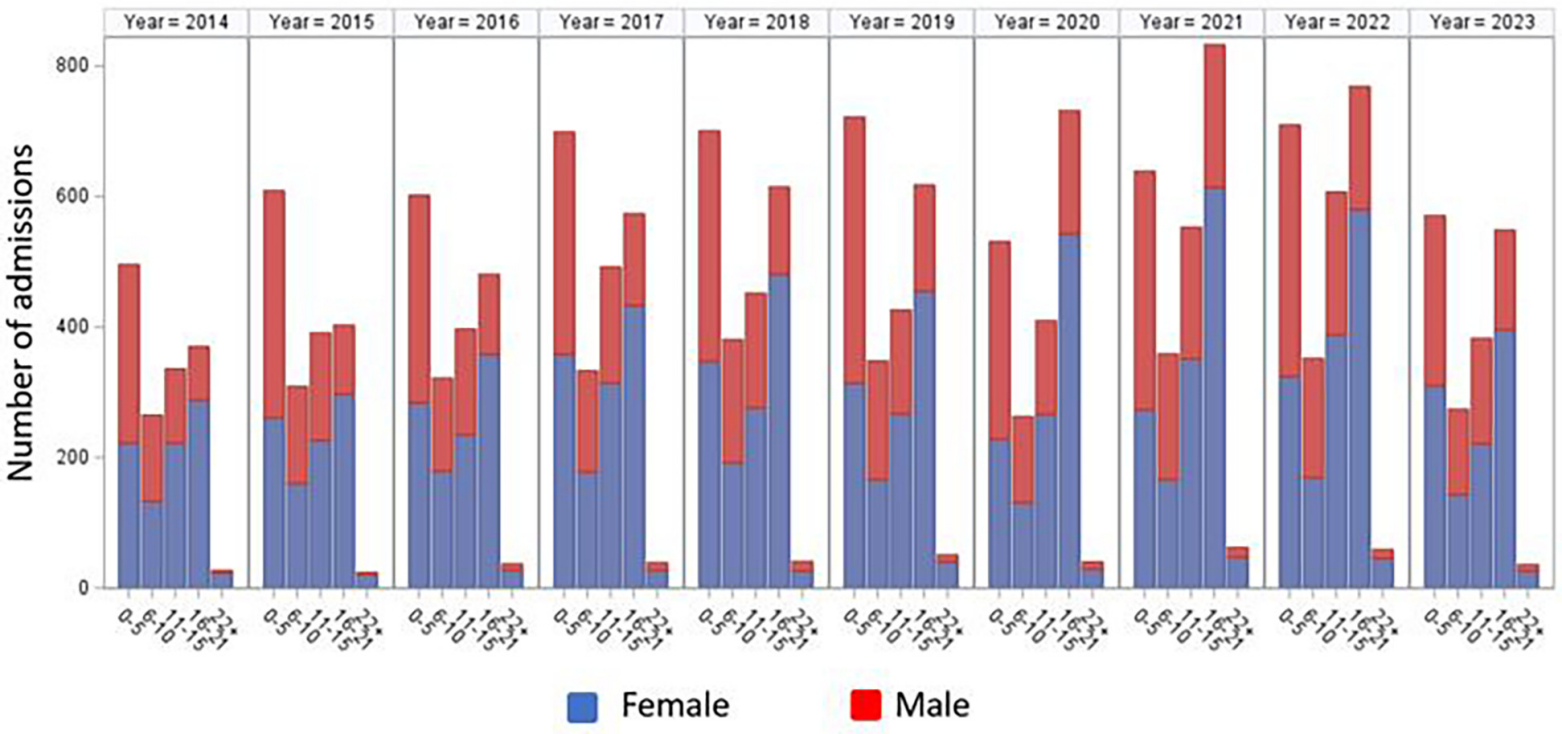
Annual admissions depicted per age group, stratified by gender.

A total of 20,293 Gastroparesis related admissions were recorded in the PHIS database between January 2014 and September 2023. The annual distribution of cases is found in Table 3. Patients' demographics are found in Table 4. Figure 2 shows the annual distribution of admissions according to age group stratified by gender. Patients younger than 5 and adolescents between 16 and 21 accounted for the highest number of admissions representing 30.9% and 29.3% respectively. Female admissions were more common, accounting for 59.4% of the total admissions. Differences in geographical distribution were evident as most admissions were seen in the South (31.5%), followed by the Midwest (28.5%). Caucasians represented 69.7% of the study population.

**Table 3:** Annual distribution of number of admissions.

Year	*n* (%)
Total	20,293 (100)
2014	1,494 (7.37)
2015	1,736 (8.55)
2016	1,839 (9.06)
2017	2,137 (10.53)
2018	2,190 (10.79)
2019	2,165 (10.69)
2020	1,976 (9.73)
2021	2,446 (12.06)
2022	2,497 (12.30)
2023 (up until September 30)	1,813 (8.93)

**Table 4:** Number of subject in each patient demographics.

Variable	Description	*N* (%)
Total		20,293 (100)
Gender	Female	12,060 (59.43)
	Male	8,233 (40.57)
Age	0–5	6,280 (30.95)
	6–10	3,206 (15.80)
	11–15	4,447 (21.91)
	16–21	5,944 (29.29)
	22–25	416 (2.05)
Race	Caucasian	14,147 (69.71)
	African American	2,604 (12.83)
	Asian	423 (2.08)
	Others/Mixed	2,002 (9.87)
	Unknown	1,117 (5.51)
Region	Northeast	3,606 (17.77)
	South	6,388 (31.48)
	West	4,517 (22.26)
	Midwest	5,782 (28.49)
Priority of admission	Emergent/Urgent	16,952 (83.54)
	Elective	3,136 (15.45)
	Other	205 (1.01)

### Characteristics of admissions

The median length of stay of GP related admissions was 6 days and the mean was 13.6 days. The longest admission lasted 653 days. Emergent and urgent admissions represented 83.6% of the admissions, and ICU stay was required in 17.2% of the admissions. There was a 0.6% mortality rate.

### Primary source of payment

**Figure 3:**
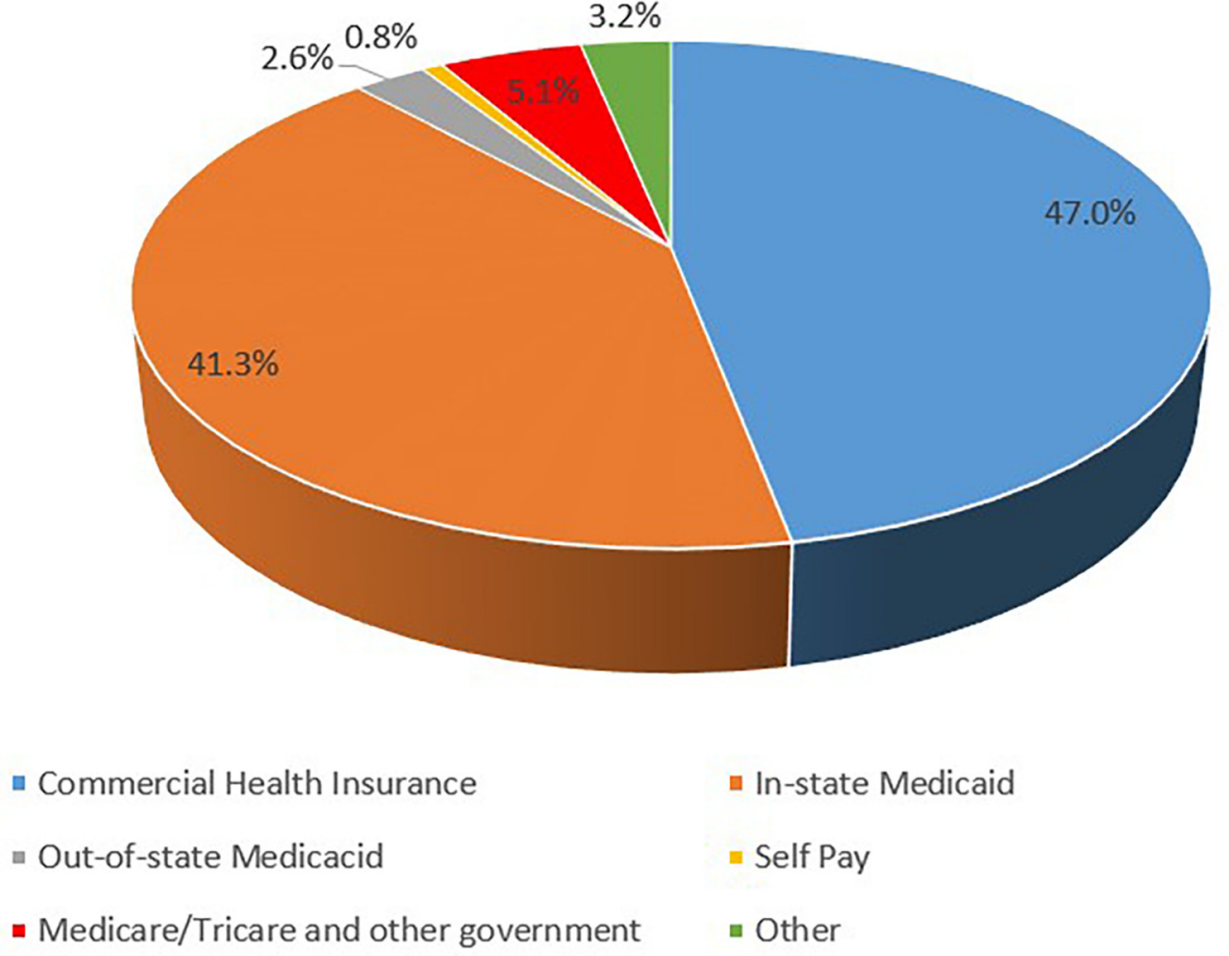
Sources of primary healthcare coverage for pediatric patients with gastroparesis.

Most admissions were covered by insurance rather than self-pay as 49.1% were covered by private insurance and 47.6% covered by public insurance. In-State Medicaid was the primary source of payment in 41.3% of the admissions. Out-of-State Medicaid covered 2.6% of the admissions, representing 5.9% of all Medicaid covered admissions. Commercial health insurances were the primary source of payment for 47.0% of the admissions. Figure 3 depicts the percentile distribution of the primary source of healthcare coverage for pediatric patients with GP.

### Cost of admissions

**Figure 4:**
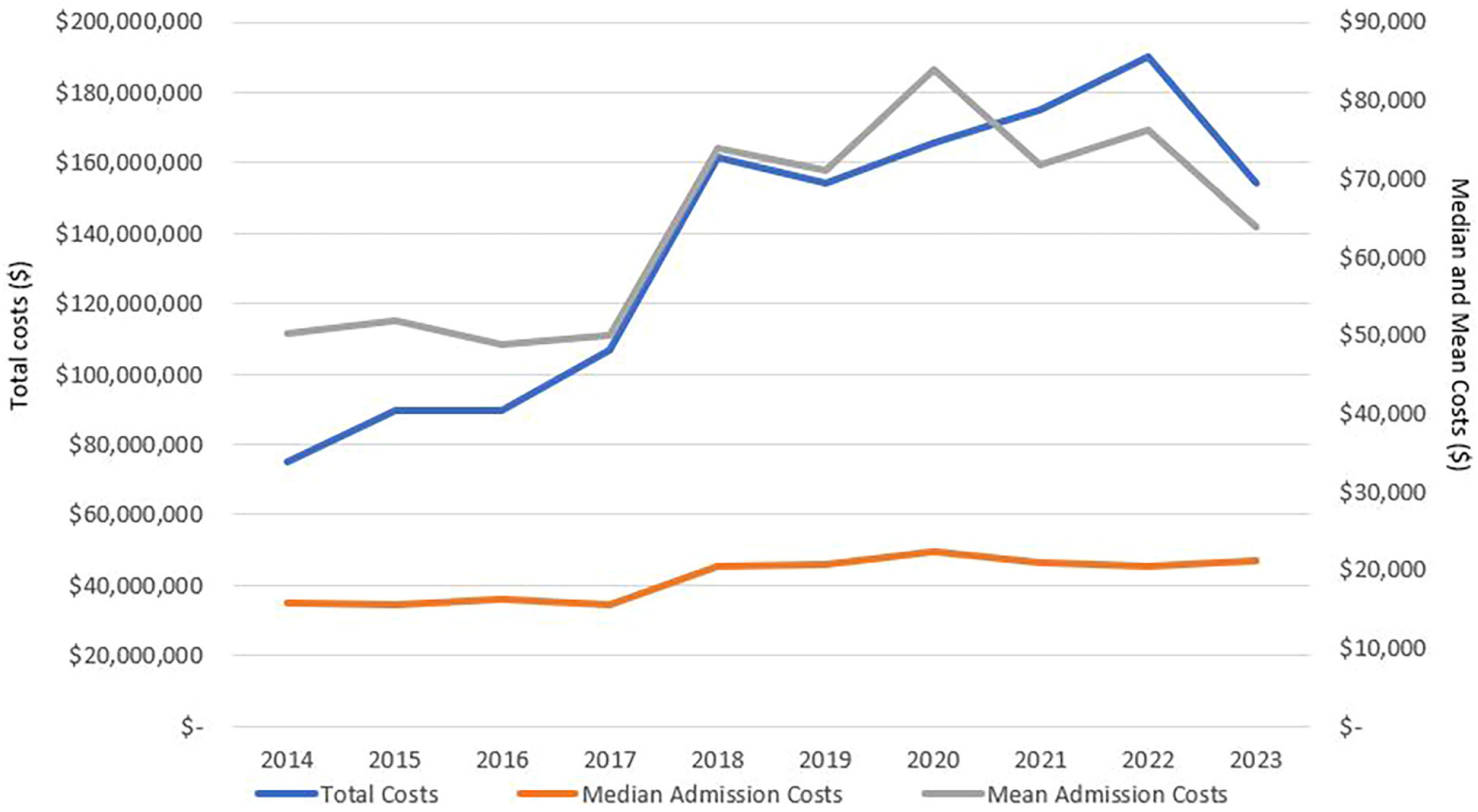
Annualized total, mean, and median costs of pediatric gastroparesis admissions.

**Figure 5:**
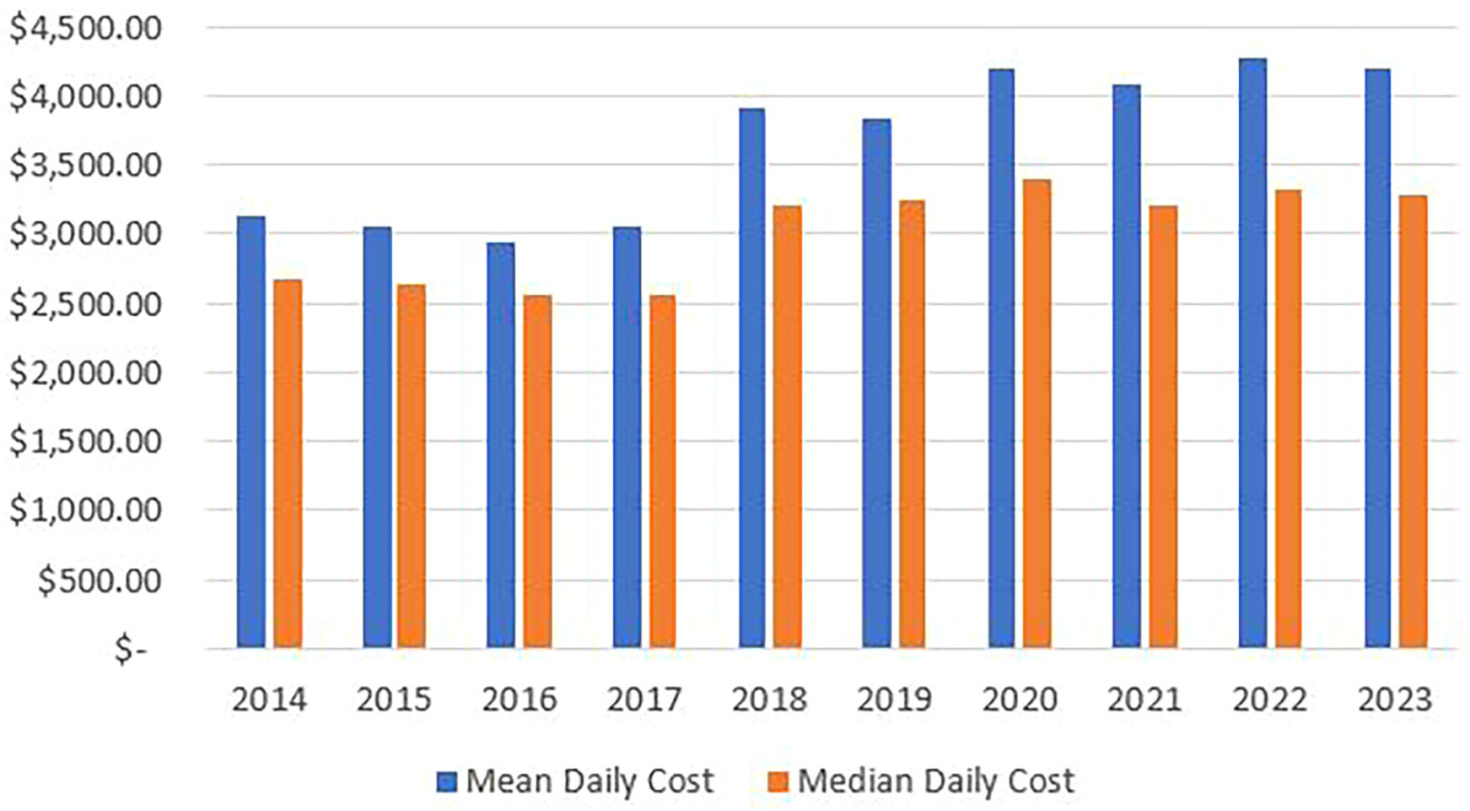
Mean and median daily costs arranged by years.

Our data showed that gastroparesis admissions in the listed children's hospitals presented significant economic burden on the healthcare systems costing more than $1.3 billion over the study duration. In 2014, the total costs almost reached $75 million, and this number increased annually to peak at $190 million in 2022. Indeed, the total, mean, and median annual costs of admission have been increasing over the past decade as seen in Figure 4. Mean and median annual costs were highest in 2020 as the mean reached $83,842 and the median reached $22,351. Moreover, mean and median daily costs followed similar trends to those of the annual admission costs, as seen in Figure 5.

### Categorical distribution of costs

**Figure 6:**
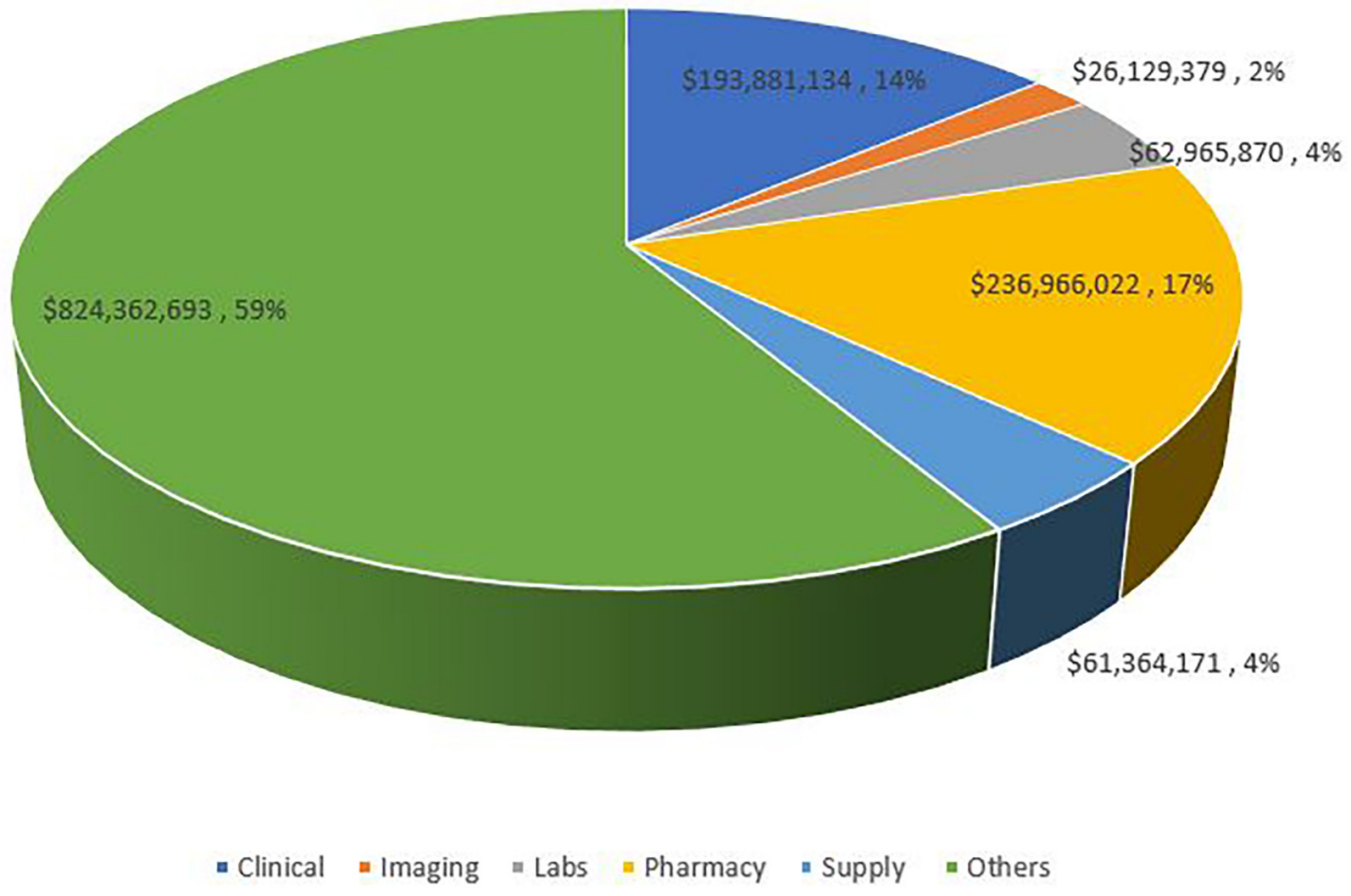
Distribution of admission costs per category of expenses. Others include nursing units, psychiatric treatment units, ancillary services to treatment, dietary services, social services, discharge planning services, and other ancillary services.

**Figure 7:**
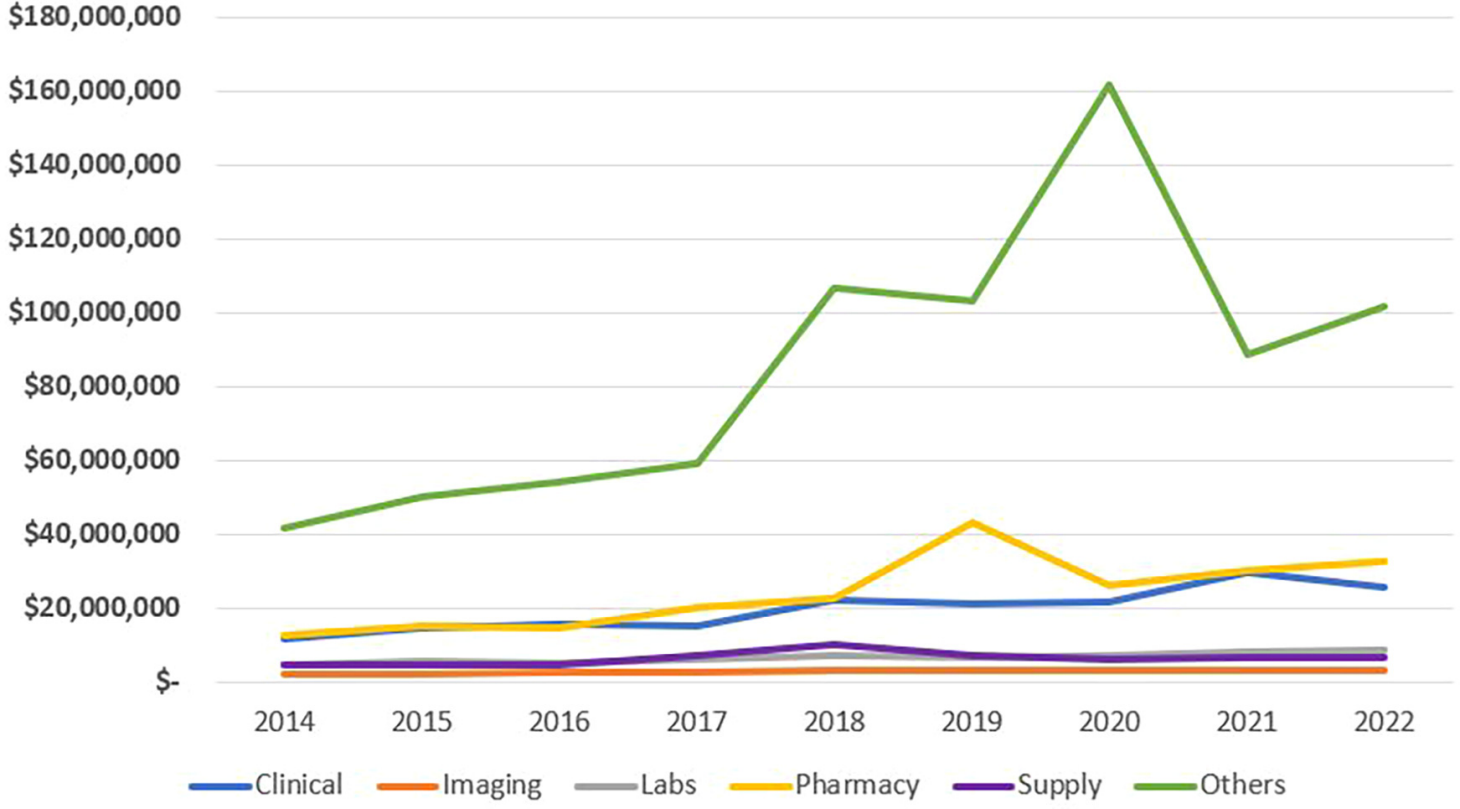
Total annual cost per category of expenses.

As seen in Table 5 and Figure 6, 58.6% of total costs were labeled as “other” costs, followed by Pharmacy, clinical categories, lab and supplies with minimal difference, and lastly by imaging., “*The cost under the ‘Other’ category is mostly attributable to floor services, including nursing units, psychiatric treatment units, ancillary treatment services, dietary services, social services, discharge planning services, and other related ancillary care.*”*.* Annual changes in the categorical distribution of total costs can be seen in Figure 7. There was a marginal increase in all categories between 2014 and 2017. After 2017, clinical, pharmacy, and other categories began to increase at a higher rate. Pharmacy costs peaked in 2019 reaching a 3.5-fold increase whereas clinical costs peaked in 2021 exceeding a 2.5-fold increase and the “others” peaked in 2020 reaching a 3.9-fold increase all compared to the costs in 2014. represented 71.9% of total costs in 2020.

**Table 5:** Categorical distribution of pediatric gastroparesis admissions cost.

Time	All	2014	2015	2016	2017	2018	2019	2020	2021	2022	2023
Total costs											
Clinical	193,881,134	11,462,357	14,816,863	15,846,089	15,296,763	22,238,516	21,337,996	21,521,325	29,530,224	25,449,141	16,381,860
Imaging	26,129,379	2,014,194	2,119,240	2,452,089	2,375,420	2,865,979	2,891,141	2,872,625	3,224,089	3,241,534	2,073,068
Labs	62,965,870	4,606,343	4,606,343	5,009,854	5,968,546	6,933,605	6,517,065	6,959,410	8,061,749	8,651,299	4,715,679
Pharmacy	236,966,022	12,360,661	15,325,159	14,391,538	21,124,696	22,821,047	25,861,243	25,861,243	30,181,991	32,627,744	20,077,058
Supplies	61,364,171	4,780,180	4,800,031	4,577,630	6,877,498	10,227,872	7,205,841	6,068,576	6,618,318	6,560,791	3,647,434
Other	624,362,693	41,563,596	50,209,410	54,301,918	59,161,538	106,437,229	102,860,631	161,845,177	88,822,332	101,745,608	57,415,254
Mean cost											
Clinical	9,554	7,672	8,535	8,617	7,158	10,155	9,856	10,891	12,073	10,192	9,036
Imaging	1,288	1,348	1,221	1,333	1,112	109	1,335	1,454	1,318	1,298	1,143
Labs	3,103	3,083	3,193	2,724	2,793	3,166	3,010	3,522	3,296	3,465	2,601
Pharmacy	11,677	8,274	8,828	7,826	9,417	10,421	19,951	13,088	12,339	13,067	11,074
Supplies	3,024	3,200	2,765	2,489	3,218	4,670	3,328	3,071	2,706	2,627	2,012
Other	40,623	27,820	28,922	29,528	27,684	48,601	47,511	81,905	36,313	40,747	31,669
Median cost											
Clinical	1,146	900	959	1,028	1,059	1,143	1,254	1,305	1,267	1,188	1,242
Imaging	404	402	401	470	372	351	394	525	407	388	389
Labs	850	787	819	823	764	833	836	1,023	928	860	815
Pharmacy	1,254	1,137	1,066	1,201	1,087	1,279	1,355	1,565	1,342	1,222	1,326
Supplies	143	326	229	240	175	175	174	224	67	23	0
Other	12,551	10,745	10,768	11,601	11,256	12,341	12,915	14,161	13,589	13,450	14,422

### Regional distribution of costs

“*In examining the rising costs of GP admissions, we hypothesized that these costs would correlate with the cost of living in the geographic region where each hospital is located*.” The cost of admission varies between the 4 national regions defined by PHIS. The West and Midwest had the highest mean annual admission cost per admission with $72,520 and $70,171 respectively; the South had a mean of $61,909 while the Northeast had a mean admission cost of $54,012. As for the median annual admission cost per admission, the Midwest ranked highest with $21,459, followed by the West with $19,989, then the Northeast had the third highest median with $17,578, and finally the South with $16,532. Daily median and mean regional costs followed similar trends to the total admission costs. Mean and median daily and total admission costs were highest in 2020 in all 4 regions.

**Figure 8:**
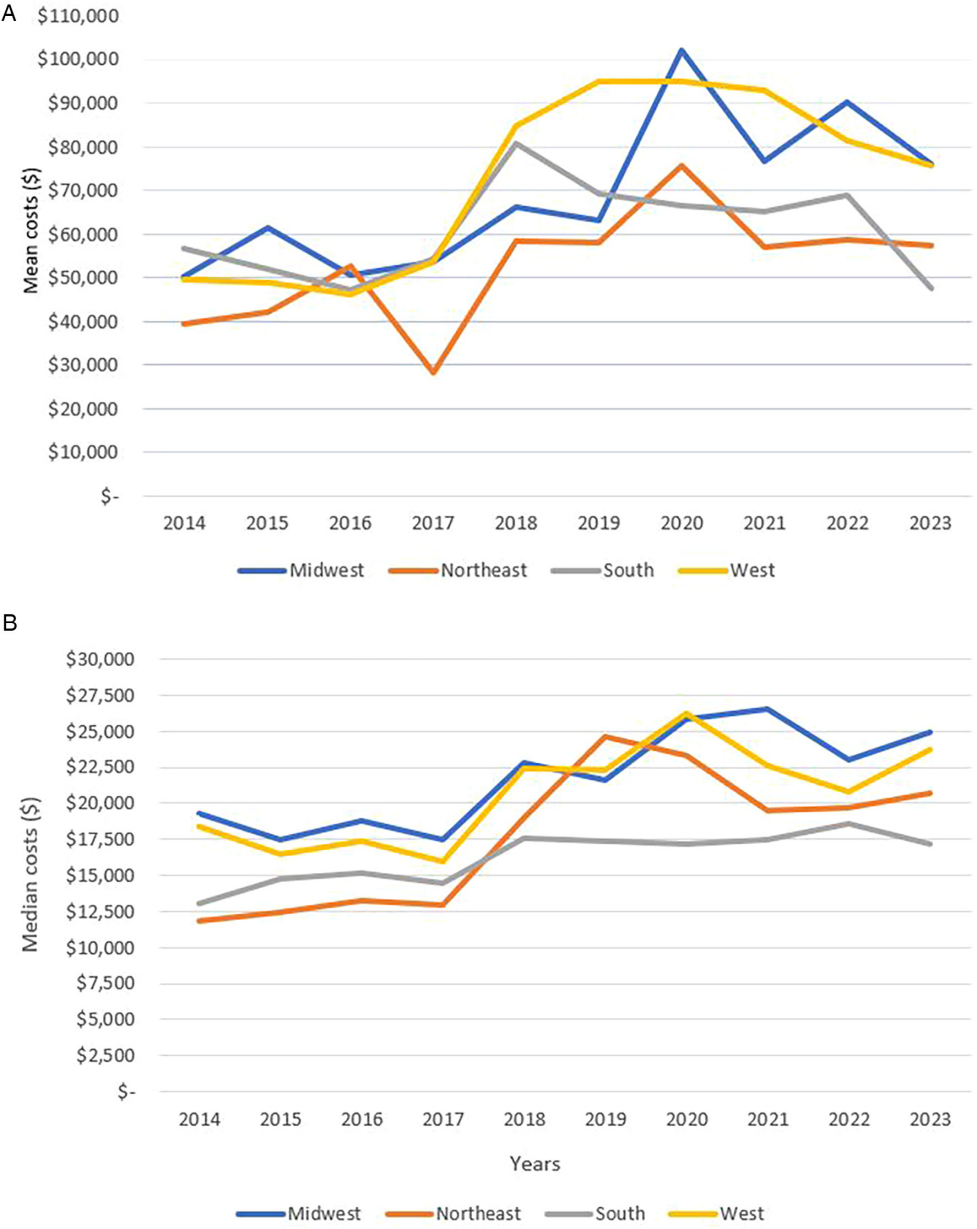
**(A)** Trend of mean annual cost of pediatric gastroparesis admissions in the 4 USA geographic regions. **(B)** Trend of median annual cost of pediatric gastroparesis admissions in the 4 USA geographic regions.

**Figure 9:**
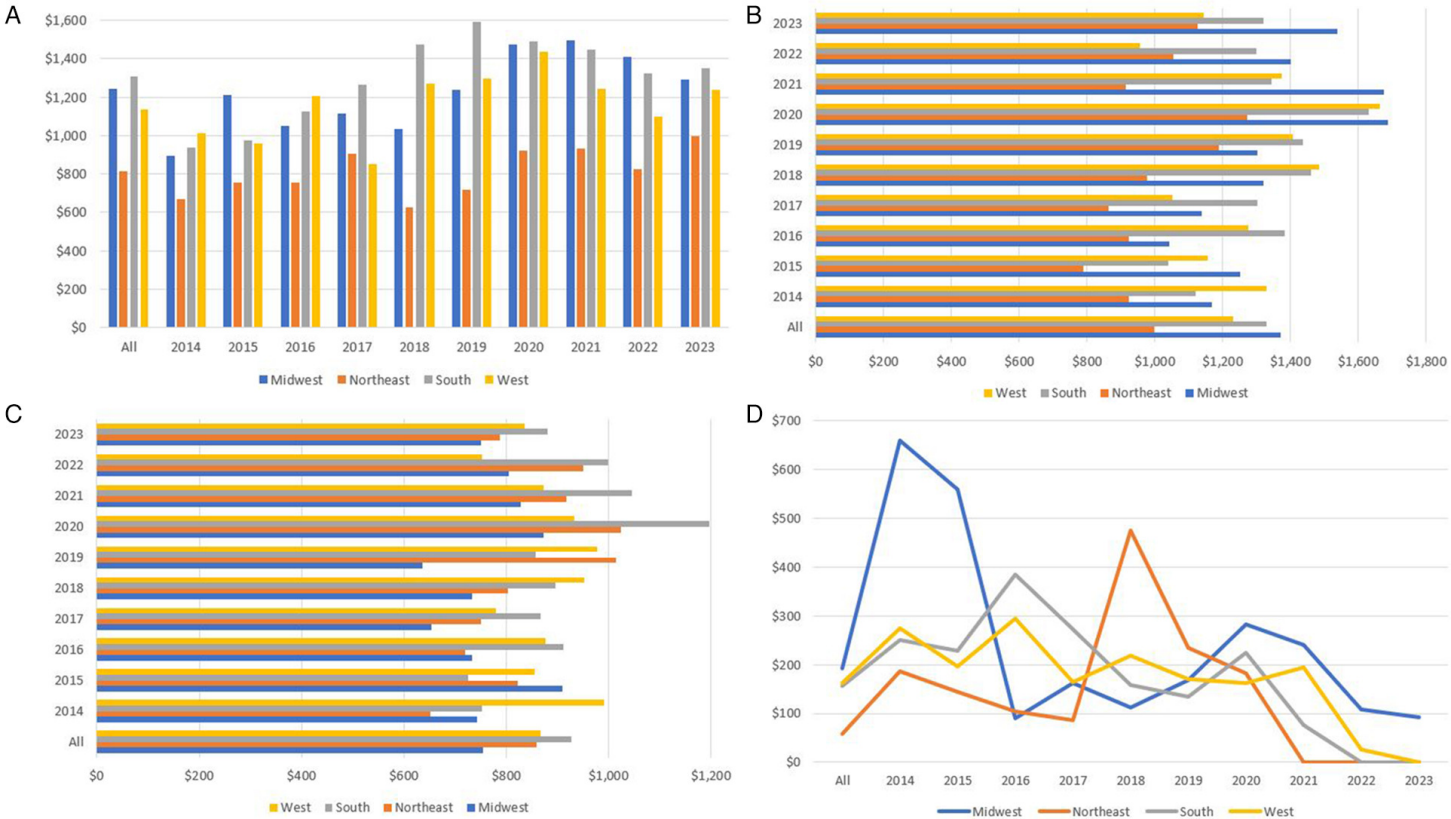
**(A)** The different geographic distribution of median clinical expenses per year. **(B)** The different geographic distribution of median pharmacy expenses per year. **(C)** The different geographic distribution of median lab expenses per year. **(D)** The different geographic distribution of median supplies expenses per year.

We also compared the categorical cost distribution over the 4 regions. Total, mean, and median categorical costs had similar trends in all 4 regions as the national distribution. However, in the Midwest, pharmaceutical expenses represented a higher proportion, and “other” expenses represented a lower proportion of the total and mean costs. The South had a higher proportion of clinical expenses compared to the other regions. Another difference was that total and mean supplies costs were highest in the South; however, this was not the case for the median supplies cost. The South is the only region where total and mean supplies costs were higher than laboratory costs. Median lab costs were higher in the Northeast region as it is the third highest cost in the Northeast surpassing the median clinical costs. Figure 8 shows the mean and median annual cost of pediatric GP admissions in the four USA geographic regions. Figure 9 shows the annual median differences of the clinical, pharmacy, lab, and supplies categories in the four geographic regions.

### Enteral feeding

We found from the database that malnutrition was a common indication for Gastroparesis admission. Using malnutrition and weight loss ICD-9 and ICD-10 codes, we found that 15.7% of admissions had malnutrition as a diagnosis and 15.1% of admissions had weight loss as a diagnosis.

Feeding supplementation was commonly seen in Pediatric GP admissions between 2014 and September 2023.15.6% of admissions required Nasogastric tube (NG), 24.6% of admissions received feeding via a feeding tube placed surgically (SFTS); surgical feeding tube supplementation as coded by PHIS. Gastrostomy tube (G-tube) is reported in 40.7% of admissions and 10.0% of admissions reported having a jejunostomy tube (J-tube) with possible overlap.

Overall, we saw a relative decrease in the coding of the used supplies between 2014 and 2023. For example, in 2014, 35.0% of patients had a code of SFTS, while only 15.8% and 14.7% of patients had such a code SFTS in 2022 and 2023 respectively. Also, we found that the code used for NG tube was at 28.9% at its highest in 2014, decreasing to 7.2% in 2022 and 2023. The highest proportion of patients with G-tube code was in 2017 as 45.5% of patients had a G-tube present. This number then decreased to reach 35.8% in 2022% and 37% in 2023. However, there were minimal differences in the proportion of patients with a J-tube code over the years; however, nationally the proportion of admissions coding for J-tube were higher between 2014 and 2017 compared to 2018–2023. The highest percentage of patients with J-tube was in 2016 with 11.6%. Unfortunately, searching PHIS data for PN code did not produce any concise data as a result we did not have any reportable data on PN use in this population.

The total cost related to the supplies category increased from around $4.8 million in 2014 to reach a peak of around $10.2 million in 2018 and then decreased to around $6.6 m in 2022 as seen in Figure 7. Mean admission supplies costs followed similar trends to the total annual supplies costs and also peaked in 2018 reaching $4670. Conversely, the median admission supplies cost was highest in 2014, $326, with this number decreasing annually to reach a value of $23 in 2022 and even $0 in 2023. There was an exception to this trend as the cost increased from $174 in 2019 to $224 in 2020.

**Figure 10:**
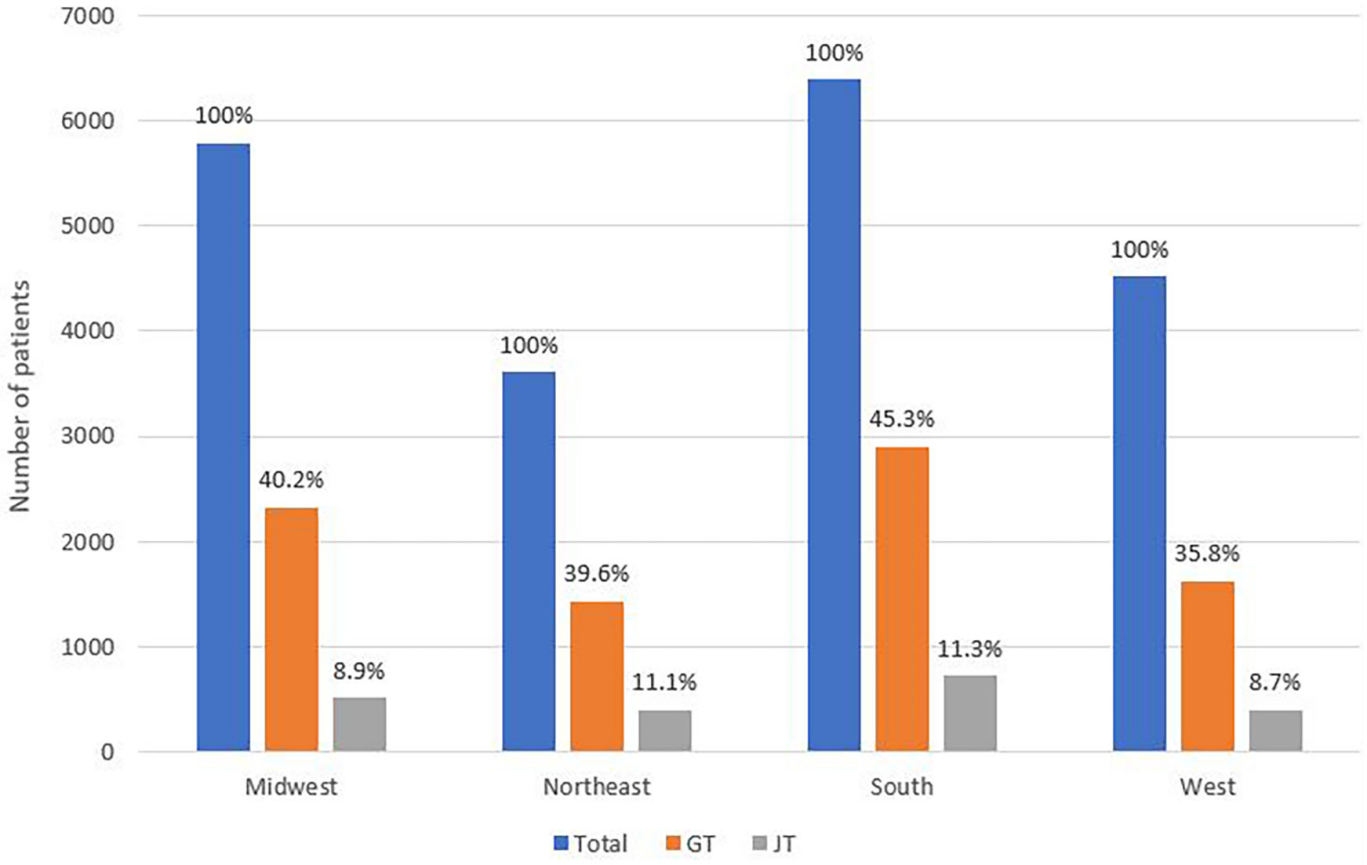
Comparison of the proportion of patients requiring J-tube and G-tube to the total number of admissions in each of the 4 regions.

Considering that all feeding tubes are part of the supplies category, we then compared the supplies usage between the different regions. The South region had the highest use of NG tubes with 23% of admissions followed by the Northeast with 15.4%, then the West with 14.1%, and finally the Midwest with 8.8%. Patients with G-tube were seen mostly in the South and least commonly in the West. There was a similar trend of increase between 2014 and 2017 followed by a gradual decrease after in all 4 regions. Patients with J-tube were seen mostly in the South and Northeast regions and less commonly in the West and Midwest. Similar to the national trend, patients with J-tube were coded for more commonly between 2014 and 2017 than 2018–2023 with the exception of the Midwest where the proportion slightly increased. Figure 10 provides a comparison of the percentage of patients requiring J-tube, or G-tube to the total number of admissions in each of the 4 regions.

## Discussion

This study describes the admission characteristics and the associated costs of children with gastroparesis over the last decade in the United States of America. We found that the total, mean, and median annual cost of pediatric GP admissions have increased more than two-fold compared to the decade prior. We stratified the cost over 6 different inpatient expense categories and analyzed the cost differences between the 4 US geographic regions. “Based on our findings and the PHIS data analysis, we propose several actionable recommendations for clinical practice and the management of pediatric gastroparesis. Our data suggest that comorbid conditions significantly contribute to prolonged length of stay and increased admission costs. Attention should also be given to patients’ nutritional status in the outpatient setting to help prevent malnutrition, which often necessitates extended hospitalization.”

Interestingly, we found that children under the age of five were more likely to require prolonged admissions. This highlights the need for early and proactive outpatient management in younger children with gastroparesis. Clinicians may consider earlier use of prokinetic agents, intra-pyloric botulinum toxin injections, or transpyloric feeding as part of disease management in this age group. “Finally, as expected, patients with a higher number of complex chronic conditions had significantly longer hospital stays, further increasing the overall cost of GP-related admissions.” To our knowledge, this is the first study reporting the distinct categories leading to the high costs of GP admissions. This is also the first study to report the prevalence of using enteral feeding in patients with gastroparesis.

We found a high level of complexity in pediatric GP admissions. High associated costs are likely partially reflective of this. There was an overall high requirement of surgically placed feeding tube supplementation (SFTS) affecting 24.6% of pediatric gastroparesis admissions. Further, many patients had a concomitant diagnosis of malnutrition and also a high number of complex chronic conditions. Patients tended to be admitted in a non-elective manner. They required ICU at high rates and had high mortality rates as well. Reflecting this complexity, we found that the length of stay (LOS) is prolonged 5.5 times in children with GP compared to the LOS in the whole pediatric population (23). Further reflecting their complexity, is the rate at which pediatric GP patients require out of state healthcare.

The percentage of Medicaid admissions covered in out of state hospitals for children with GP was 5.9%. There is minimal data about out-of-state Medicaid spending; nevertheless, in 2020, the Medicaid and CHIP Payment and Access Commission (MACPAC), a federal government agency, issued a report about the out-of-state spending in the fiscal year 2013. They found that 1.9% of all Medicaid hospital stays were out-of-state with disabled children being the group with the highest requirement for these stays at 4.1% (24). Using this data, pediatric GP patients had a 3.1-fold and near a 1.5-fold increased requirement for out-of-state admissions compared to the general population group and to the disabled children group respectively. These data may demonstrate that there is a higher need in pediatric GP patients compared to other pediatric patients to go to a tertiary care center in a different state to receive the level of required care. It may also reflect the high acuity, non-elective nature of pediatric GP admissions. It therefore is important to promote standardized guidelines and educate providers across all types of pediatric centers so patients can receive optimal care, minimizing urgent hospitalizations and also remain closer to home for care and early intervention.

Malnutrition diagnosis is found to be associated with a longer stay and higher probability of readmission in the general population regardless of admission diagnosis (25). A nationwide study from 2016 to 2019 found that 12.5% of adult GP admissions presented with malnutrition had more complications, high mortality rates, had significantly longer duration of stay and higher costs of admissions (26). We found that pediatric GP patients had an even higher rate of malnutrition of 15.7%. As a consequence of malnutrition and the high need for nutritional rehab, there is high rate of enteral feeding in our population. Our data in pediatrics is comparable to a study done in the Netherlands on 86 adults with GP; 19 of these patients (22.1%) required a GJ tube as their symptoms were refractory to diet and prokinetics and short-term gastric rest via ND feeding (27). In this study, 84% of patients who required a GJ tube remained dependent on it after an average usage duration of 962 days (27). However, the data are heterogenous in the adult literature. A different study on 971 adults in the US from the National Institute of Diabetes and Digestive and Kidney Disease (NIDDK) gastroparesis registry, found only 26 patients (2.7%) required enteral feeding at any point during their study (28).

Jejunal feeds are known to improve symptoms and reduce admission requirements through decreasing the frequency of acute symptom exacerbation (29). In our study, the percentage of admissions coding for SFTS and supplies usage kept on decreasing after 2018 even with the pandemic. We believe that these findings are consistent with what is reported previously that placement of J-tube provides access to improved nutrition and decrease the need for hospitalization.

The cost of pediatric GP admissions has more than doubled since 2014 accounting for more than $190 million in 2022. This increase in cost is precedented as sixfold and even tenfold increases have been seen in longitudinal epidemiologic studies (2, 7). GP related Emergency department visits numbers and costs have more than doubled between 2006 and 2013 (30). Although a nationwide study found that the mean admission costs were increasing from 1997 to 2013, this difference was not found in the pediatric population between 2004 and 2013 (2, 7). Hence, in that study, the increase in the number of admissions might have had the largest effect on total costs in that pediatric population.

The increase in total costs of pediatric gastroparesis admissions in our study is multifactorial, and not only attributable to the number of admissions. Total costs increased by more than 2.5-fold, whereas the number of admissions increased by 1.7-fold. Both mean and median costs increased by around a 1.5-fold between 2014 and 2022 and 2023. To better understand this increase, we looked at the changes in the expenses related to different categories. Compared to the increase in the total admission expenses, there is a relatively smaller increase in costs of the imaging, labs, and supplies categories. We hypothesize that improved national education on GP, advances in medications and technology, and wider spread standardized of GP care have led to this decrease in unnecessary imaging, labs, and supplies usage. Furthermore, the changes seen in the supply costs could be due to the decrease in the need for admission in patients already on tube feeding, which would also explain the decrease in the proportion of admitted patients dependent on feeding supplementation in our study. This finding aligns with that placing a G or J tube will facilitate out of the hospital nutritional rehabilitation and lessen the need for admission. On the other hand, increases in the clinical, pharmacy, and other categories have had a much larger contribution to the increase in costs having each increased by at least 2-fold. The increase in the professional categories, clinical and pharmacy, might indicate an increase in severity as these patients are requiring more care and either more medication or more parenteral feeding, which is accounted for in the pharmacy category expenses. The increase in the other category, which was the greatest increase, reflects a major increase in hospital expenses such as the room itself. In addition, a recent study found that in 2019, up to 30% of the total national health expenditures were attributed to administrative expenses estimating up to $1 trillion. The primary drivers of these administrative expenses were billing and coding costs, physician administrative activities, and insurance administrative costs (31, 32). Moreover, another recent study compared 2022 data about healthcare systems between major wealthy countries (United States of America, Switzerland, Germany, Netherlands, Sweden, Belgium, France, Canada, Australia, Ireland, United Kingdom, Japan, and Italy). They found that the United States had the highest healthcare spending per capita of all these countries with the spending reaching almost double the average of all countries. The US also had by far the highest administrative cost which was almost five times the average of the countries studied (33).

**Figure 11:**
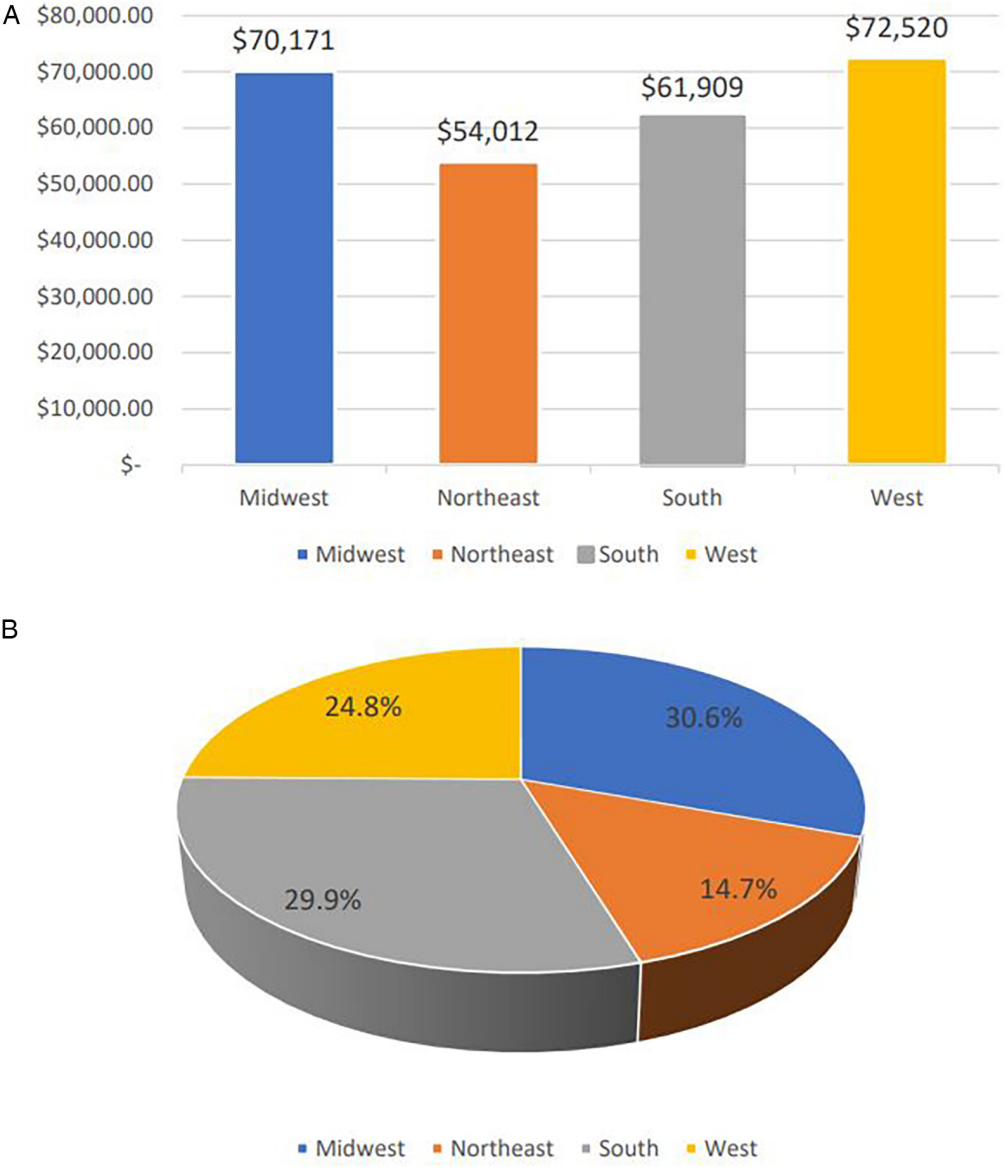
**(A)** Average cost of single admission per geographic region across the 10 years period of time. **(B)** National financial burden of pediatric gastroparesis admissions over the 10 years study duration proportionally presented per geographic regions.

We found however that the number of admissions plays a major role in driving the varying total cost for the defined national regions. Figure 11A depicts the mean annual cost of a single pediatric GP admission for each region, while Figure 11B shows the proportional representation of the national financial burden of pediatric GP admissions for the four geographic regions. The South represented a larger proportion of the total costs, while the Northeast represented a smaller proportion of the total costs compared to their respective mean admission cost due to the difference in the number of admissions recorded in the PHIS database.

We believe that these descriptive findings underscore the importance of further investigation, particularly in children under five years of age with gastroparesis. Future research should aim to identify the specific factors contributing to higher admission costs in this population. Institutional databases may offer more granular data, enabling targeted analyses of variables such as nutritional status, coexisting mental conditions, and disease complexity scores—each of which appears to play a significant role in prolonged hospital stays and increased costs associated with GP admissions.

### The effect of COVID-19

The COVID-19 pandemic had significant effects on the economy and healthcare systems worldwide (10, 34). It additionally posed a significant financial strain at the individual level (35).

Advanced age and female gender were seen to be independently associated with developing Long-COVID (36). Studies reporting data on Long COVID in pediatric patient up to 6 months after the infection show a wide incidence range however the estimate of most studies fell in a range of 10%–20% (37). In fact, a 2 years longitudinal cohort study following up pediatric patients aged 11–17 found that 7.2% out of 12,632 patients developed long COVID symptoms for the entire duration of the study with the older children having the symptoms more commonly (38).

A recent meta-analysis of studies done on children and adolescents found that out of 1,2424 pediatric patients who survived a COVID-19 infection, 23.36% developed Long-COVID with at least 1 persistent symptom lasting between 3 months and up to more than a year (39). In that study, gastrointestinal symptoms were seen in as high as 11.87% of the total patients and included abdominal pain, diarrhea, and weight loss as the most common presenting symptoms (39).

In our recently reported pediatric GP epidemiologic study (40), we found an increase in adolescent admissions aged between 16 and 21 post-COVID. We also noted a significant increase of 9.04% in the monthly number of admissions. Post-pandemic annual costs have a noticeable increase in 2020 with the highest mean and median costs recorded during that year. The increase in total costs has been persistent as the highest annual cost was in 2022. Even though complexity indicators such as proportion of ICU admission rate, mortality length of stay, and priority of admission were comparable throughout the duration of the study without significant difference after the pandemic.

### Limitations

To our knowledge, this is the first paper to describe the categorical distribution of cost and feeding supplementation in the pediatric GP admissions. In this study, we retrieved data about cost of GP admissions in children and described its distribution into different categories. However, we were forced to limit our analysis to the major expense categories defined by PHIS because of their grouping and hence are unable to analyze the actual cost in the subcategories. As noticed, we pulled data on tube feeding from discharge diagnosis and it is possible that data was missed to incomplete documentation. For this same reason, we were not able to pick up and report accurate data on PN usage in this population and other forms of enteral feeding supplementation “*The subcategorization of costs would have provided particularly relevant data, especially in 2020, when the ‘Other’ category accounted for 71.9% of total costs*.”

Additional limitations with this study are that we were only able to use data from 42 pediatric hospitals as there is no national registry and that we were not able to track patients who traveled to tertiary care centers and used out of state resources. These 42 hospitals are not equally divided into the 4 regions which limits our ability to accurately compare admission regional cost variations.

To conclude, we found that the cost of pediatric gastroparesis admissions has been increasing since 2014 and the costs have been affected by the COVID-19 pandemic. The increase is multifactorial but includes an increase in the number of admissions and an increase in the clinical, pharmacy, and other categories (floor services: nursing units, psychiatric treatment units, ancillary services to treatment, dietary services, social services, discharge planning services, and other ancillary services). The increase in costs has been seen throughout the different regions of the United States of America. Finally, we have seen that most severity indices are elevated in the GP population, including feeding tube supplementation although this requirement may have contributed to better outpatient management and a lower need for hospitalization. Areas of future research could include studying specific factors affecting GP admissions and contributing to the costs and the implications for pediatric healthcare.

## Data Availability

The datasets presented in this article are not readily available because The Pediatric Health Information System database used restricts sharing data especially if the person requesting data is not from a PHIS contributing hospital. Requests to access the datasets should be directed to Children's Hospital Association, analytics.support@childrenshospitals.org.
